# 24-h urinary sodium excretion and the risk of adverse outcomes

**DOI:** 10.1080/07853890.2020.1780469

**Published:** 2020-06-30

**Authors:** Matti A. Vuori, Kennet Harald, Antti Jula, Liisa Valsta, Tiina Laatikainen, Veikko Salomaa, Jaakko Tuomilehto, Pekka Jousilahti, Teemu J. Niiranen

**Affiliations:** a Division of Medicine, University of Turku and Turku University Hospital, Turku, Finland; b Department of Public Health Solutions, Finnish Institute for Health and Welfare (THL), Helsinki, Finland; c Institute of Public Health and Clinical Nutrition, University of Eastern Finland, Kuopio, Finland; d Joint Municipal Authority for North Karelia Social and Health Services (Siun sote), Joensuu, Finland; e Department of Public Health, University of Helsinki, Helsinki, Finland; f Diabetes Research Group, King Abdulaziz University, Jeddah, Saudi Arabia

**Keywords:** Cardiovascular disease, coronary heart disease, stroke, heart failure, diabetes, sodium

## Abstract

**Aims:**

The objective was to evaluate whether sodium intake, assessed with the gold standard 24-h urinary collections, was related to long-term incidence of death, cardiovascular disease (CVD) and diabetes mellitus (DM).

**Methods:**

A cohort of 4630 individuals aged 25–64 years collected 24-h urine samples in 1979–2002 and were followed up to 14 years for the incidence of any CVD, coronary heart disease (CHD), stroke, heart failure (HF) and DM event, and death. Cox proportional hazards models were used to estimate the association between the baseline salt intake and incident events and adjusted for baseline age, body mass index, serum cholesterol, prevalent DM, and stratified by sex and cohort baseline year.

**Results:**

During the follow-up, we observed 423 deaths, 424 CVD events (288 CHD events, 142 strokes, 139 HF events) and 161 DM events. Compared with the highest quartile of salt intake, persons in the lowest quartile had a lower incidence of CVD (hazard ratio [HR] 0.70; 95% confidence interval [CI], 0.51–0.95, *p* = .02), CHD (HR 0.63 [95% CI 0.42–0.94], *p* = .02) and DM (HR 0.52 [95% CI 0.31–0.87], *p* = .01). The results were non-significant for mortality, HF, and stroke.

**Conclusion:**

High sodium intake is associated with an increased incidence of CVD and DM.

## Key message

Compared with the individuals with the highest salt intake, persons with the lowest had 1.5 times lower incidence of cardiovascular disease (CVD) and coronary heart disease, and almost twice the lower incidence of diabetes mellitus.

## Introduction

The association between high sodium intake and elevated blood pressure (BP) has been established in numerous studies [1]. However, the role of excess sodium as a risk factor for cardiometabolic disease, and particularly what may be the optimal amount of sodium intake, remain debated. In addition, the association between sodium intake and risk of certain health outcomes such as heart failure (HF) and diabetes mellitus (DM) have been scarcely studied.

Several prior adequately sized studies [2–4] on the association between salt intake and incident cardiometabolic disease have relied on sodium intake estimates derived from spot urine samples. Spot urine samples are less difficult to collect when conducting population studies, but single, or preferably even multiple 24-h urine collections are regarded as the gold standard for assessing habitual sodium intake [5]. Spot urine samples tend to overestimate sodium intake in the low end, underestimate it in the high end, are susceptible to systematic error, and are not recommended by The International Consortium for Quality Research on Dietary Sodium [6]. Many studies have demonstrated a direct association between 24-h urine sodium excretion and adverse health outcomes [6–9]. Studies using inaccurate methods such as spot or overnight urine collections have, however, presented no significant associations, an inverse association between sodium excretion and mortality, or a direct association with coronary heart disease (CHD) in women only [1–3].

Due to the conflicting results and limitations of many earlier studies, the association between sodium intake and the risk of cardiometabolic diseases requires further elucidation. Therefore, we carried out a comprehensive study aiming at assessing in a large, randomly selected population sample whether sodium intake, properly assessed with 24-h urinary collections, was associated with the incidence of death, CVD including its main subcategories, and DM during a long-term follow-up.

## Methods

### Study sample

Finland is a unique country where sodium intake using 24-h urinary excretion at the population level has been systematically assessed in several successive surveys since 1979. The present study used data from up to 8990 participants of these population-based samples comprising people aged 25–64 years at baseline who participated in the North Karelia Salt Project in 1979 [7], WHO FINMONICAstudiesin1982, 1987 or the National FINRISK Study in 2002 [8] and provided a 24-h urinary collection.

The North Karelia Project was a pioneering lifestyle study in a Finnish county with very high cardiovascular mortality and morbidity. The Salt project was a substudy of this. In the baseline survey of the North Karelia Salt Project in 1979, a random sample of 2487 individuals were drawn from the National Population Register who were invited to fill in a lifestyle questionnaire and attend a physical examination. Those attending the physical examination were instructed to provide a 24-h urinary collection. In total, 1593 participants provided complete data with 24-h urine collections. We excluded individuals reporting incomplete collection and those aged 14–24 years to consider a common age range for all cohorts (Supplementary Figure S1).

The FINMONICA and FINRISK comprise a series of population surveys organised by the Finnish Institute for Health and Welfare that have been carried out every 5 years since 1982 in four different geographical regions of Finland. We considered participants of the 1982 and 1987 FINMONICA studies and the 2002 FINRISK Study (all these survey cohorts are later on called as the FINRISK surveys) [10]. For each of these surveys, a sample stratified by sex and 10-year age group from the population aged 25–64 years were randomly drawn from the national population register and invited to take part in the study. The participants filled in a lifestyle questionnaire following the extended WHO MONICA protocol [9] and underwent physical examination, including blood sampling, anthropometric and BP measurements. At each study, randomly chosen sub-sample of the participants were also asked to collect a 24-h urine specimen (Supplementary Figure S1). A total of 1382, 1151, and 909 individuals provided complete 24-h urine collections in 1982, 1987, and 2002, respectively.

A flow chart of the sample selection is provided in the Supplementary Figure S1. We excluded duplicate participations (*n* = 29, the earlier was excluded), individuals with missing data (*n* = 16), and individuals aged <25 years (*n* = 236) resulting in 4632 individuals included in the present analyses (Supplementary Figure S1). Patients with prevalent CVD (*n* = 115), CHD (*n* = 70), DM (*n* = 79), and HF (*n* = 29) at baseline were excluded from the analyses related to each corresponding outcome (Supplementary Figure S1).

All participants gave informed consent for their data to be used in scientific research purposes with anonymity. The Finnish Ethics committee has approved the study protocol and this study complies with the Declaration of Helsinki.

We adhered to the STROBE protocol (Strengthening the Reporting of Observational Studies in Epidemiology) [11] for reporting observational studies.

### Covariates

At the baseline examinations, systolic and diastolic BP, height and weight were measured by a nurse trained for the survey methods. Blood samples were drawn after a minimum of 4-h fast for the determination of serum lipids. BMI was defined as weight (kg) divided by height (m) squared. Smoking was defined as self-reported daily use of tobacco products during the last six months (data not available for the 1979 survey). Prevalent DM was defined as a register-based diagnosis of DM before baseline using the same criteria as for incident DM (see *Follow-up and outcomes*). A 24-h urine collection was performed to assess urinary sodium, potassium and creatinine excretion. The collection day was Sunday in most cases. The participants were not informed about the purposes of the urine collection in order to avoid bias from reducing salt intake before the urine collection. People who reported an incomplete 24-h collection or with urine volumes less than 1000 ml were excluded. According to an external quality assurance programme organised by Lab quality (Helsinki, Finland), the bias of the sodium assay method was 1.4% (SD 0.55, *n* = 12) in 1979, 4.0% (SD 2.08, *n* = 10) in 1982, 1.9% (SD 1.81, *n* = 10) in 1987 when measurements were performed using a flame photometer, and decreased to 0.2% (SD 1.85, *n* = 12) in 2002, when an ion-selective electrode was used. Details of the urine collections have been described earlier [12].

### Follow-up and outcomes

The primary outcomes in our study were death, and incidence of CVD, CHD, HF, stroke, and DM. The participants were followed up through the National Causes of Death Register (CDR), the Finnish Hospital Discharge Register (FHDR) [10] and the Drug Reimbursement Register for up to 14 years after the baseline assessment.

The CHD event was defined as the International Classification of Diseases 10th edition (ICD-10, Finnish version) codes I20-I22 and ICD-8/9 codes 410 or 4110 in the FHDR for nonfatal cases; or as the ICD-10 codes I20-I25, I46, R96, R98 or the ICD-8/9 codes 410-414 in the CDR for fatal ones.

HF was defined as the ICD-10 codes I50, I110, I130, I132 or the ICD9 codes 4029B, 4148, and 428, or the ICD-8 codes 42700, 42710 and 428 in the FHDR.

Stroke was defined as the ICD-10-codes I61 and I63-64 (but not I636, excluding subarachnoid haemorrhages and venous occlusions, but including intracerebral haemorrhages) or the ICD-9 codes 430-314,330A, 4331A, 4339A, 4340A, 4341A, 4349A, and 436, or the ICD-8 codes 431 (except 43101, 43191) 433, 434, 436.

DM was defined as the ICD-10 codes E10-E14 or the ICD-8/9 code 250 in the FHDR or CDR. Because ICD-8 only had one code for diabetes, we, too, were forced to combine the subtypes of DM.

CVD was defined as the onset of CHD, stroke, or HF.

The FHDR and CDR have been validated for diagnoses of CHD, myocardial infarction, stroke and HF and shown to provide accurate diagnostic information [10,13–15].

### Statistical methods

We divided the participants into quartiles according to their 24-h urinary sodium excretion. The characteristics of the study sample by sodium excretion quartiles were compared using ANOVA with equal variance assumption for continuous variables and chi-squared tests with continuity correction for categorical variables. Cox proportional hazards models were used to estimate the risk of adverse health outcomes at different levels of sodium excretion. The proportional hazards assumption of the models was tested using Schoenfeld residuals. The fourth (highest) quartile had the largest number of events and was used as the reference category in all analyses to maximise statistical power. We also used restricted cubic splines with four knots to model the continuous, non-linear association between urinary sodium excretion and adverse health outcomes. For these analyses the median sodium excretion (170.6 mmol/day, corresponding to 10.0 g NaCl/day) was used as the reference level with hazard ratio 1.0, bypassing the categorical quartiles. We included age, survey year, sex, serum total cholesterol, prevalent DM and BMI as covariates in analyses for mortality and CVD events, and age, survey year, sex, serum total cholesterol and BMI as covariates for the DM analyses. All analyses were stratified by age and survey cohort year, i.e., a separate hazard function was estimated for each combination of sex and cohort in the statistical model. We included systolic BP as a covariate only in a sensitivity analysis to avoid over-adjustment bias, i.e. controlling for an intermediate variable on a causal path from exposure to outcome. In addition, we included smoking as a covariate for the 1801 participants of the 1982, 1987 and 2002 cohorts with data available in a separate analysis. The effect of cohort was also studied in a separate subgroup analysis. All analyses were performed using R v.3.5.1.

## Results

Selected characteristics of the study sample are presented in Table 1. The mean age at baseline was 45.4 years and 51.5% were women. The proportion of women decreased, while BMI, serum total cholesterol, and systolic BP increased with the increasing quartiles of sodium excretion. The mean sodium excretion was 183 mmol/day (corresponding to 10.7 g NaCl/day), decreasing with the successive survey cohort years (Table 1).

**Table 1.** Characteristics by quartiles of 24-hour urinary sodium excretion.

Characteristic	Quartile of urinary sodium excretion				Total	*p* Value
	Q1	Q2	Q3	Q4		
*N*	1157	1159	1158	1158	4632	
Age, years	45.4 (11.7)	45.4 (11.5)	45.4 (11.4)	45.5 (10.8)	45.4 (11.4)	.99
Women, *n* (%)	812 (70.2)	703 (60.7)	548 (47.3)	324 (28.0)	2387 (51.5)	<.001
Body mass index, kg/m^2^	25.2 (4.1)	25.8 (4.1)	26.7 (4.2)	27.9 (4.4)	26.4 (4.3)	<.001
Total cholesterol, mmol/l	5.9 (1.3)	6.0 (1.2)	6.2 (1.3)	6.4 (1.3)	6.1 (1.3)	<.001
Systolic BP, mmHg	138 (21)	139 (20)	142 (21)	145 (20)	141 (21)	<.001
24-h urinary Na						
Range, mmol/day	12–127	127–171	171–224	225–685	12–685	<.001
Mean, mmol/day	97.1 (21.9)	149.0 (12.8)	196.6 (15.6)	289.5 (61.4)	183.1 (78.6)	<.001
Estimated 24-h salt (NaCl) intake						
Range, g/day	0.7–7.4	7.4–10.0	10.0–13.1	13.1–40.0	0.7–40.0	<.001
Mean, g/day	5.7 (1.3)	7.9 (0.61)	10.0 (0.6)	12.5 (0.8)	10.7 (4.6)	<.001
Examination year, n (%)						
1979	212 (18.3)	266 (23.0)	342 (29.5)	410 (35.4)	1230 (26.6)	<.001
1982	260 (22.5)	319 (27.5)	380 (32.8)	389 (33.6)	1348 (29.1)	<.001
1987	258 (22.3)	328 (28.3)	289 (25.0)	274 (23.7)	1149 (24.8)	<.001
2002	427 (36.9)	246 (21.2)	147 (12.7)	85 (7.3)	905 (19.5)	<.001

Data are presented as mean (SD) unless indicated otherwise. *p* Values for continuous variables are from regular ANOVA with equal variance assumption and for categorical variables from chi-squared tests with continuity correction. One gram of salt intake is calculated as equal of 17.1 mmol sodium excretion. BP: blood pressure; SD: standard deviation.

The individuals were followed for death for a median time period of 14.0 years accumulating 62 402 person years. During this time frame, we observed 423 deaths, 424 CVD events, 288 CHD events, and 142 strokes. In addition, 142 individuals developed DM and 139 HF (Table 2). The proportion of individuals experiencing adverse health events increased across the increasing sodium excretion quartiles, except for stroke where the nadir was seen in the second lowest quartile (Table 2).

**Table 2.** Number of incident adverse outcomes by quartiles of 24-h urinary sodium excretion.

Outcome	Quartile of urinary sodium excretion				Total	*p* value
	Q1	Q2	Q3	Q4		
Mortality						
*N*	1157	1159	1158	1158	4632	
No. overall deaths	79 (6.8)	95 (8.2)	104 (9.0)	145 (12.5)	423 (9.1)	<.001
Cardiovascular disease						
*N*	1141	1131	1126	1119	4517	
No. events	72 (6.3)	84 (7.4)	105 (9.3)	163 (14.6)	424 (9.4)	<.001
Coronary heart disease						
*N*	1151	1140	1137	1134	4562	
No. events	39 (3.4)	64 (5.6)	74 (6.5)	111 (9.8)	288 (6.3)	<.001
Stroke						
*N*	1150	1152	1154	1153	4609	
No. events	35 (3.0)	19 (1.6)	33 (2.9)	55 (4.8)	142 (3.1)	.004
Diabetes mellitus						
*N*	1145	1145	1134	1129	4553	
No. events	24 (2.1)	26 (2.3)	45 (4.0)	66 (5.8)	161 (3.5)	<.001
Heart failure						
*N*	1153	1157	1149	1144	4603	
No. events	23 (2.0)	31 (2.7)	34 (3.0)	51 (4.5)	139 (3.0)	.040

Data are presented as n (%). Individuals with a prevalent disease have been removed from the corresponding analyses. Cardiovascular disease event includes composite outcomes of coronary heart disease, stroke and heart failure. *p* Values are for chi-squared tests.

**Figure 1.**
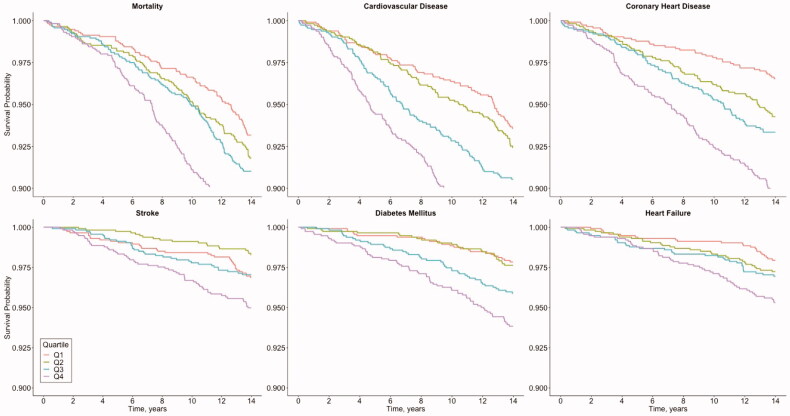
Outcome-free survival after baseline by quartiles of 24-h sodium excretion. Log rank *P* value for all outcomes < .001. Numbers of censored events and individuals at risk at each time point are provided in Supplementary Table S1.

**Figure 2.**
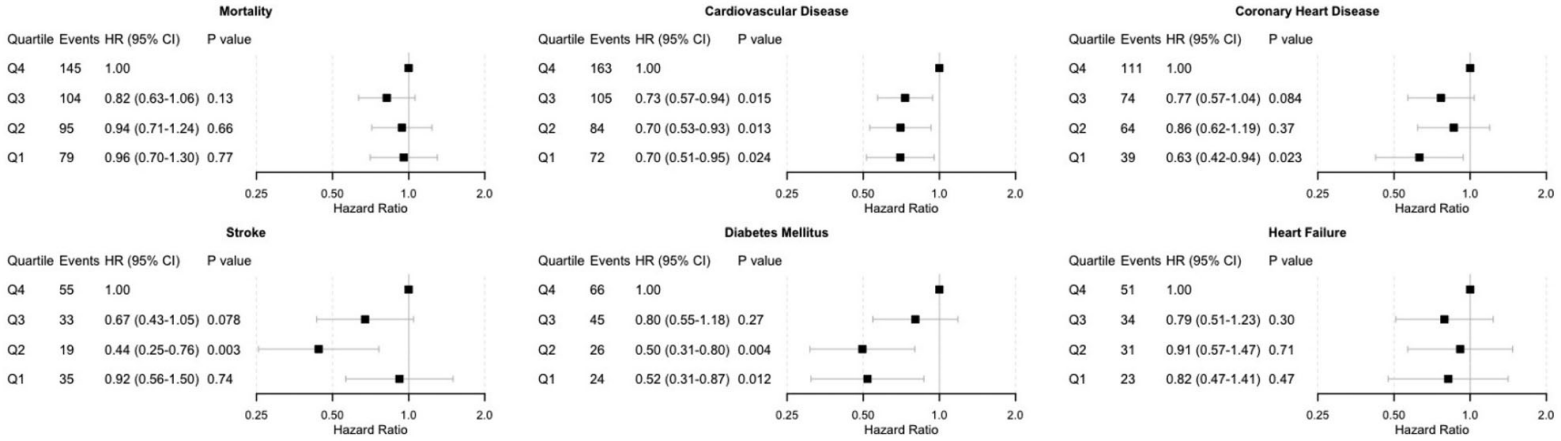
Multivariable-adjusted risk of adverse health outcomes by quartiles of 24-h urinary sodium excretion. Models are adjusted for baseline age, body mass index, cholesterol, prevalent diabetes and stratified by sex and cohort. Individuals with a prevalent disease in question at baseline have been removed from the corresponding analyses. CI: confidence interval; HR: hazard ratio.

Outcome-free survival is depicted in Figure 1with Kaplan–Meier curves. The number of censored individuals and those remaining at risk at any given time are reported in Supplementary Table S1. The unadjusted and adjusted risks of incident adverse health events in the quartiles of sodium excretion are presented in Supplementary Figure S2 and Figure 2. In the unadjusted models (Supplementary Figure S2), the risk of adverse health events was always the lowest in the first quartile of sodium excretion, except for stroke. The stroke incidence in the second quartile of sodium excretion was significantly lower than in the highest (fourth) quartile. In the multivariable-adjusted models, we observed significantly lower risks for CVD, CHD and DM in the lowest quartile of sodium excretion compared with the highest quartile (Figure 2).

**Figure 3.**
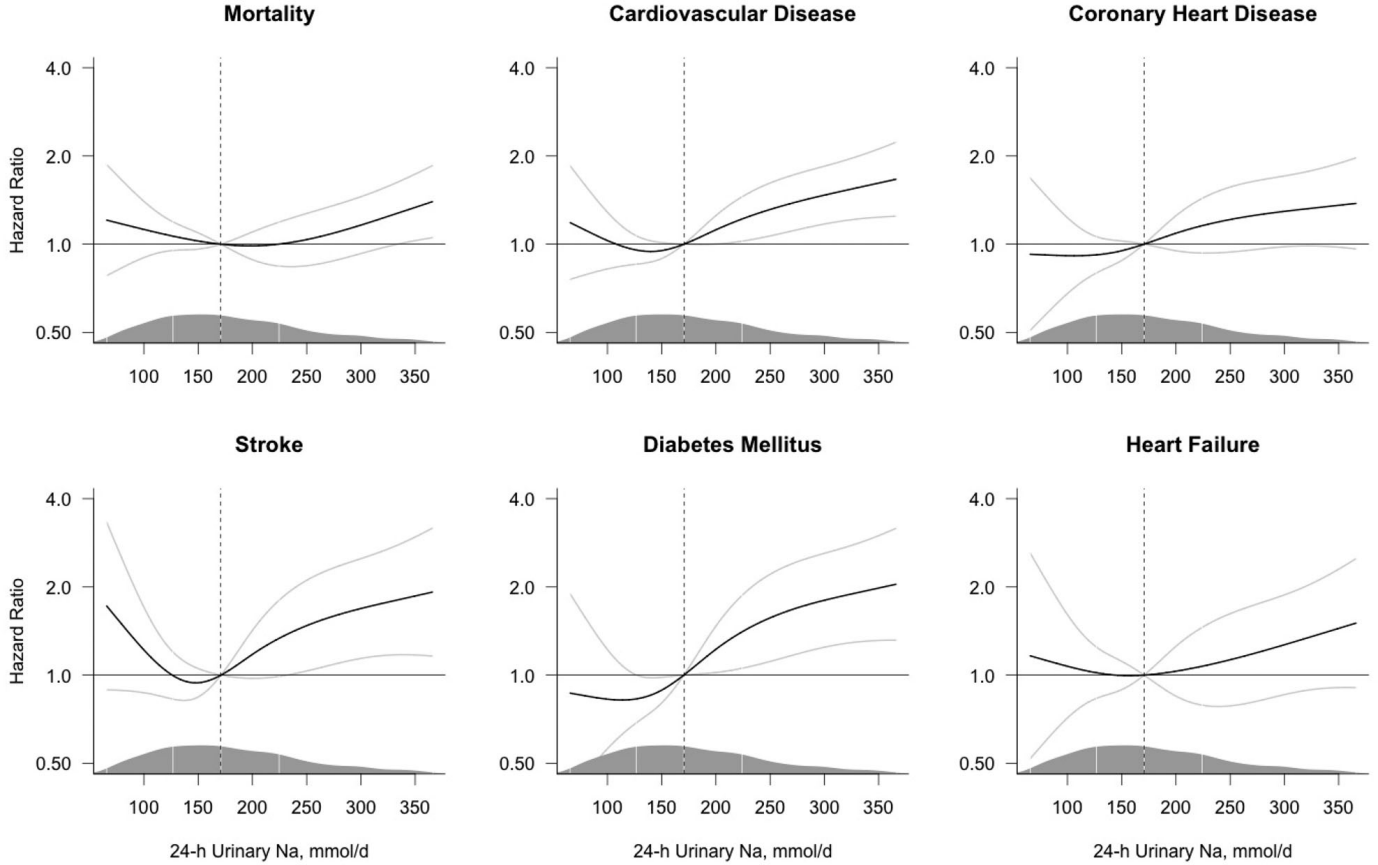
Multivariable-adjusted risk of adverse health outcomes by 24-h sodium excretion. Hazard ratios (black line) were estimated using Cox proportional hazards regression and plotted by restricted cubic splines. The models are centered at the median (dashed line, 170.6 mmol/day; hazard ratio = 1.0) with 4 knots at the 5^th^, 35^th^, 65^th^ and 95^th^ percentiles and the plot truncated at the 2.5th and 97.5th percentiles. The grey lines represent the 95% confidence interval. The models are adjusted for baseline age, body mass index, cholesterol, prevalent diabetes and stratified by sex and cohort. The density of the observations along the spline variable is marked by the mountain plot, marking the median and the 1st and 3rd quartile with vertical lines.

The unadjusted analyses of the continuous association between sodium excretion and the risk of adverse health outcomes are shown in Supplementary Figure S3; the risk of all adverse health outcomes increased with the increasing level of sodium excretion. However, the adjustment for covariates markedly attenuated these associations (Figure 3); only the risk of CVD, stroke and DM significantly increased with increasing sodium excretion, with a borderline increase in mortality. For stroke, the observed numerical increase of risk at the lower levels of sodium excretion was not statistically significant in the adjusted or unadjusted analyses.

We also performed a sensitivity analysis including smoking as a covariate in the 1801 individuals with smoking data available (Supplementary Figure S4). The results remained essentially unchanged but became somewhat stronger for DM and HF and attenuated the shape of the curve in the stroke analysis. The associations were similar when the cohorts were analysed separately and also when systolic BP was included as a covariate in the models (Supplementary Figures S5 and S6).

## Discussion

This study was carried out to elucidate whether sodium intake, measured with a single 24-hour baseline urinary collection, is associated with an increased risk of mortality and cardiometabolic adverse events during the longest follow-up thus far. We found a direct association between the 24-hour urinary sodium excretion and the incidence of CVD, CHD, stroke and DM, but not with all-cause mortality and HF. The risk of disease was lowest when sodium excretion was between 120 and 150 mmol/day, i.e. 7.0–8.8 grams of salt per day. However, as some of the cohorts were examined at a time when mean sodium intake in the Finnish population was >10 g/day [12], we refrain from making any strong recommendations on the optimal level of salt intake for CVD prevention. Even though CVD risk profiles have changed during the last 40 years due to changes in lifestyle and therapeutic modalities, CVD risk increased in all cohorts with increasing sodium excretion.

Several other cohort studies have been carried out on the relation of 24-h or overnight sodium excretion and cardiovascular mortality and morbidity (namely CHD and stroke) in Scotland, Northern Belgium, the Netherlands and the United States [16–19]. The median follow-up time in these studies ranged from 3.8 to 7.9 years. Alderman et al. reported an inverse relation with 24-h urinary sodium excretion at baseline and a subsequent myocardial infarction in hypertensive individuals in 1995 [19], which was strongly criticised for bias [20]. The Scottish Heart Health Study investigators observed a borderline inverse gradient for mortality and urinary sodium excretion, and a direct gradient for CHD in women only [16]. An inverse association of sodium excretion and CVD and mortality was surprisingly discovered in the Belgian study [17]. The results from the Netherlands reported a direct association between urinary sodium excretion and mortality [18]. Prior results from the National FINRISK Study also showed a direct, statistically significant association of 24-h urinary sodium for CVD morbidity and mortality, particularly in men [21]. The sample of this study consisted of approximately half of the participants included also in our current study, precisely 35 023 person years (56%) in contrast to 62 402 person years of follow-up in our current study. In addition to observational data that are prone to bias, some data also exist from intervention studies. In the trial by Cook et al., a reduction in 24-h urinary sodium excretion achieved by nutritional education and counselling in the intervention group resulted in a 25% lower CVD morbidity [22]. The findings on the direct association of excess sodium excretion and CVD were further supported also by a recent meta-analysis of observational studies by Poggio et al. [23]. Our results on the harmful effects of excess sodium intake on CVD and CHD support the earlier findings from the National FINRISK Study [21], the Scottish Heart Health Study [16], and the interventional trial by Cook et al. [22] Overnight urine collections have been shown to underestimate 24-h values [24], a potential reason why the Dutch study [18] investigators did not observe a correlation between urinary sodium excretion and CVD. There are also numerous potential reasons for the contradictory, inverse associations reported in the Belgian study and by Alderman [17,19]. Individuals with CVD at baseline were not excluded from the study by Alderman, leading to possible reverse causality when study participants at highest CVD risk may have been instructed to lower their sodium intake. In addition, there was evidence of systematic errors in sodium assessment in both studies and other limitations, also, which are described in detail in the meta-analysis by Cobb et al. [20].

The association of excess sodium excretion measured by 24-h urine collections and the onset of type 2 DM has been studied once by Hu et al. [25] using part of the cohort included in our present study (1935 participants of the FINMONICA 1982 and 1987 cohorts). The investigators observed a multivariable-adjusted hazard ratio of 2.05 in the highest vs. combined lower quartiles of urinary sodium excretion and type 2 DM. Our present study extends and confirms the findings of Hu et al. on the association between sodium excretion and DM by having twice the sample size, a longer follow-up, a wider sodium excretion range, and including most incident cases of DM. The exact underlying causes linking sodium excretion and incident DM remain unclear, but currently available processed foods almost without exception contain high concentrations of sodium. Thus, it is possible that sodium excretion in this case only acts as a proxy for energy intake in some individuals thereby resulting in risk of obesity, metabolic syndrome and type 2 DM. In addition, there is some evidence linking insulin resistance and hypertension-related salt sensitivity in obese individuals [26]. Although we adjusted our analyses for BMI, which would account for much of this potential collinearity, some residual confounding may still remain.

To our knowledge, the association of excess sodium intake and incident HF has not been studied before using the 24-h urinary sodium excretion data. Using spot urine samples and lifestyle questionnaires, the EPIC-Norfolk study showed a statistically significant increased risk of HF (hazard ratio 1.32 in the highest quintile compared with the second lowest quintile) in a study sample of 25,639 persons from the UK [27]. There was a marked attenuation of this association (hazard ratio, 1.21; 95% confidence interval, 0.98–1.49) after adjusting for hypertension, a major risk factor for HF. This might have been an over-adjustment, since high salt intake is a risk factor for hypertension; thus, hypertension is not a confounding factor in this respect.

An inverse relation with stroke and urinary sodium excretion using multiple 24-h collections was reported in an earlier study by Kieneker et al. [28]. On the contrary, another study reported that stroke patients who had not reduced their salt intake previously were more likely to suffer from a recurring lacunar stroke [29]. Our study demonstrated a mild J-curve for the association between salt excretion and stroke risk, which was different from other CVD outcomes where associations were increasing monotonously. However, the increased risk of stroke in the lowest level of sodium excretion was greatly attenuated in the sensitivity analysis with smoking included as a covariate, demonstrating the crucial role that smoking plays as a risk factor for stroke. In addition, the J-shaped association may attenuate when additional 24-h collections taken at different time points are used for assessing sodium intake [6].

In addition to difficulties with an accurate measurement of sodium intake and study settings, sodium metabolism is very convoluted. Many aspects of it still remain elusive due to challenges related to variation in dietary sodium intake and urinary sodium excretion, and total body sodium content and its regulation [5]. These challenges could also contribute to the varying results on the relation between sodium intake and CVD outcomes in the previous studies. Measuring body sodium homeostasis is relatively inaccurate as sodium excretion has a wide day-to-day and within-day variability and because sodium intake and excretion are not in balance within a 24-h timeframe. Furthermore, recent evidence suggests that total body sodium is not regulated tightly within narrow limits and sodium may be instead stored within the body up to a certain limit [30]. Data obtained in the closed environment of the Mars500 study [31] revealed a fairly long infradian rhythmic of approximately six days of the daily urinary excretion with considerable day-to-day within-person variability even with a fixed daily sodium intake. These findings together with the previous results [24] suggest that possibly a single 24-h urine collection, not to mention a spot urine sample, is too inaccurate for assessing habitual sodium intake of an individual. Unfortunately, 24-h urine collections also have other limitations such as challenges related to incomplete urine collections and variations in sodium excretion caused by everyday life situations. In addition, obtaining even single 24-h urine collections is often burdensomefor the study participants.

In addition to difficulties in measuring body sodium homeostasis, research on the effects of sodium on health has numerous pitfalls. These limitations, such as systematic error in sodium assessment, potential reverse causality, inadequate adjustment for confounders, imbalance across study groups, etc., are detailed in the meta-analysis of prior studies on urinary sodium and CVD outcomes by Cobb et al. [20]. We avoided many of these limitations in our study; reverse causality was addressed by excluding individuals with relevant prevalent disease conditions at baseline, we adjusted for all relevant covariates, including age, BMI, serum cholesterol, smoking and prevalent DM and stratified the analyses by sex and cohort year, and performed sensitivity analyses with additional covariates. Individuals with self-reported incomplete urine collections were excluded from our analyses. However, several limitations are still present. Assessing medication or kidney function has not been part of the study protocol and might affect the results. Regression dilution bias in sodium assessment may have remained in our study as the participants provided only a single 24-h urine specimen. In addition, the sample collection day of Sunday might have had an effect on the mean sodium levels due to differences between weekend and weekday eating habits. However, the daily amount of sodium consumed is reflected in urinary sodium excretion mainly several days later [31] and Sunday collections can be expected to mirror also weekday sodium consumption to some extent. Furthermore, we still expect the rank order of the participants to remain mainly the same as it would have been with a random collection day. Sunday was chosen simply because it was the easiest day of the week to collect urine for a large cohort of people, and thus would result in more collections returned. Furthermore, advances in medicine during the 30-year-period that our study covers predispose the earliest participants to cardiovascular conditions in a different manner than the participants in the more recent surveys. During the study period there have been some changes in diagnostic criteria for CVD and DM, but these have not affected differentially people at different levels of sodium intake at baseline. Also, we don’t know the number of people with insulin-deficient or type 1 DM in the study because of a single mutual code in ICD-8 , although statistically most of our cases would have type 2 DM. In addition, the Hospital Discharge and Drug Reimbursement Registers have not been validated for diabetes diagnoses. However, since people with diabetes have had free-of-charge glucose-lowering drug treatment once registered in the Drug Reimbursement Register, virtually all diabetic patients have used this privilege.

Excess sodium intake at the population level is very common. Identification of individuals who would benefit most from avoiding excess sodium is regarded as one of the crucial steps in reducing CVD morbidity and mortality [23]. This study provides additional evidence on the harmful health effects of excess sodium intake in the general population.

## Supplementary Material

Supplemental Material

## Data Availability

The data is available on permission from the THL Biobank (https://thl.fi/en/web/thl-biobank).
