# Supplementary material for: 24-h urinary sodium excretion and the risk of adverse outcomes
Source: Ann Med. 2020 Jun 30;52(8):488–96. doi: 10.1080/07853890.2020.1780469 (PMC7877963; doi:10.1080/07853890.2020.1780469)
Supplement: Supplemental Material [file IANN_A_1780469_SM6400.docx]

**24-h Urinary Sodium Excretion and the Risk of Adverse Outcomes**

Matti A. Vuori^a,b^, Kennet Harald^b^, Antti Jula^b^, Liisa Valsta^b^, Tiina Laatikainen^b,c,d^, Veikko Salomaa^b^, Jaakko Tuomilehto^b,e,f^, Pekka Jousilahti^b^, Teemu J. Niiranen^a,b^

**Supplementary Files**

**Figure legends for supplementary files**

**Table S1.** Numbers of individuals at risk and censored events at each time point. Numbers are reported as individuals at risk [censored events] for Kaplan-Meier curves in **Figure 2**.

**Figure S1.** Flow chart depicting the study sample selection. Abbreviations: HR, hazard ratio; CI, confidence interval.

**Figure S2.** Unadjusted risk of adverse health outcomes by quartiles of 24-h urinary sodium excretion. Abbreviations: HR, hazard ratio; CI, confidence interval.

**Figure S3.** Unadjusted risk of adverse outcomes by 24-hour sodium excretion. Hazard ratios (black line) were estimated using Cox proportional hazards regression and plotted by restricted cubic splines. The models are centered at the median (dashed line, 170.6 mmol/day; hazard ratio = 1.0) with 4 knots at the 5^th^, 35^th^, 65^th^ and 95^th^ percentiles and the plot truncated at the 2.5th and 97.5th percentiles. The grey lines represent the 95% confidence interval. The density of the observations along the spline variable are marked by the mountain plot, marking the median and the 1st and 3rd quartile with vertical lines.

**Figure S4.** Multivariable-adjusted risk of adverse health outcomes by 24-hour sodium excretion with smoking included as a covariate. Hazard ratios (black line) were estimated using Cox proportional hazards regression and plotted by restricted cubic splines. The models are centered at the median (dashed line, 170.6 mmol/day; hazard ratio = 1.0) with 4 knots at the 5^th^, 35^th^, 65^th^ and 95^th^ percentiles and the plot truncated at the 2.5th and 97.5th percentiles. The grey lines represent the 95% confidence interval. The models are adjusted for baseline age, body mass index, cholesterol, prevalent diabetes, smoking and stratified by sex and cohort. The density of the observations along the spline variable are marked by the mountain plot, marking the median and the 1st and 3rd quartile with vertical lines. Analyses are limited to n=1801 individuals with available smoking data (1979 cohort removed).

**Figure S5.** Multivariable-adjusted risk of adverse health outcomes by 24-hour sodium excretion with baseline systolic blood pressure included as a covariate. Hazard ratios (black line) were estimated using Cox proportional hazards regression and plotted by restricted cubic splines. The models are centered at the median (dashed line, 170.6 mmol/day; hazard ratio = 1.0) with 4 knots at the 5^th^, 35^th^, 65^th^ and 95^th^ percentiles and the plot truncated at the 2.5th and 97.5th percentiles. The grey lines represent the 95% confidence interval. The models are adjusted for baseline age, body mass index, cholesterol, prevalent diabetes, smoking and stratified by sex and cohort. The density of the observations along the spline variable are marked by the mountain plot, marking the median and the 1st and 3rd quartile with vertical lines.

**Figure S6.** Multivariable-adjusted risk of cardiovascular disease by 24-hour sodium excretion, subgroup analysis by cohort year. Hazard ratios (black line) were estimated using Cox proportional hazards regression and plotted by restricted cubic splines. The models are centered at the median (dashed lines; hazard ratio = 1.0) with 4 knots at the 5^th^, 35^th^, 65^th^ and 95^th^ percentiles and the plot truncated at the 2.5th and 97.5th percentiles. The grey lines represent the 95% confidence interval. The models are adjusted for baseline age, body mass index, cholesterol, prevalent diabetes, smoking and stratified by sex and cohort. The density of the observations along the spline variable are marked by the mountain plot, marking the median and the 1st and 3rd quartile with vertical lines.

**Table S1 – Risk table.**

|  | Time (years) | | | | | | | |
| --- | --- | --- | --- | --- | --- | --- | --- | --- |
|  | 0 | 2 | 4 | 6 | 8 | 10 | 12 | 14 |
| Mortality |  |  |  |  |  |  |  |  |
| Q1 | 1157 (0) | 1151 (0) | 1146 (0) | 1138 (0) | 1124 (0) | 1118 (0) | 1103 (0) | 0 (1078) |
| Q2 | 1159 (0) | 1151 (0) | 1142 (0) | 1135 (0) | 1119 (0) | 1103 (0) | 1087 (0) | 0 (1064) |
| Q3 | 1158 (0) | 1149 (0) | 1142 (0) | 1129 (0) | 1114 (0) | 1099 (0) | 1074 (0) | 0 (1054) |
| Q4 | 1158 (0) | 1147 (0) | 1135 (0) | 1113 (0) | 1085 (0) | 1055 (0) | 1038 (0) | 0 (1013) |
| Cardiovascular Disease |  |  |  |  |  |  |  |  |
| Q1 | 1141 (0) | 1130 (4) | 1117 (7) | 1105 (10) | 1089 (17) | 1079 (21) | 1061 (30) | 0 (1069) |
| Q2 | 1131 (0) | 1117 (7) | 1104 (11) | 1090 (12) | 1069 (19) | 1052 (26) | 1036 (32) | 0 (1047) |
| Q3 | 1126 (0) | 1114 (3) | 1096 (4) | 1066 (11) | 1041 (18) | 1021 (25) | 993 (35) | 0 (1021) |
| Q4 | 1119 (0) | 1096 (5) | 1061 (11) | 1030 (18) | 1006 (24) | 966 (36) | 939 (43) | 0 (956) |
| Coronary Heart Disease |  |  |  |  |  |  |  |  |
| Q1 | 1151 (0) | 1142 (5) | 1132 (8) | 1121 (14) | 1109 (22) | 1100 (26) | 1082 (37) | 0 (1112) |
| Q2 | 1140 (0) | 1126 (7) | 1115 (11) | 1103 (12) | 1085 (20) | 1069 (28) | 1056 (35) | 0 (1076) |
| Q3 | 1137 (0) | 1124 (5) | 1111 (8) | 1090 (17) | 1070 (25) | 1049 (35) | 1023 (46) | 0 (1063) |
| Q4 | 1134 (0) | 1114 (7) | 1084 (14) | 1059 (25) | 1035 (35) | 997 (53) | 976 (63) | 0 (1023) |
| Stroke |  |  |  |  |  |  |  |  |
| Q1 | 1150 (0) | 1141 (5) | 1132 (9) | 1125 (13) | 1107 (26) | 1100 (32) | 1085 (44) | 0 (1115) |
| Q2 | 1152 (0) | 1144 (8) | 1133 (17) | 1125 (23) | 1106 (37) | 1090 (52) | 1073 (64) | 0 (1133) |
| Q3 | 1154 (0) | 1146 (7) | 1136 (13) | 1114 (25) | 1096 (38) | 1079 (50) | 1052 (72) | 0 (1121) |
| Q4 | 1153 (0) | 1141 (9) | 1122 (18) | 1093 (37) | 1066 (59) | 1034 (82) | 1010 (96) | 0 (1098) |
| Diabetes Mellitus |  |  |  |  |  |  |  |  |
| Q1 | 1145 (0) | 1138 (6) | 1129 (10) | 1122 (17) | 1109 (29) | 1098 (34) | 1079 (49) | 0 (1121) |
| Q2 | 1145 (0) | 1136 (6) | 1127 (14) | 1120 (21) | 1103 (34) | 1084 (49) | 1065 (63) | 0 (1119) |
| Q3 | 1134 (0) | 1125 (7) | 1112 (13) | 1094 (25) | 1075 (38) | 1055 (49) | 1028 (67) | 0 (1089) |
| Q4 | 1129 (0) | 1113 (8) | 1098 (18) | 1069 (37) | 1039 (58) | 1006 (80) | 982 (93) | 0 (1063) |
| Heart Failure |  |  |  |  |  |  |  |  |
| Q1 | 1153 (0) | 1146 (8) | 1136 (19) | 1127 (36) | 1113 (54) | 1108 (77) | 1092 (92) | 0 (1093) |
| Q2 | 1157 (0) | 1147 (6) | 1136 (11) | 1126 (20) | 1108 (35) | 1093 (48) | 1074 (68) | 0 (1115) |
| Q3 | 1149 (0) | 1137 (8) | 1127 (14) | 1114 (20) | 1095 (34) | 1081 (45) | 1050 (58) | 0 (1126) |
| Q4 | 1144 (0) | 1130 (6) | 1117 (11) | 1093 (18) | 1065 (30) | 1035 (35) | 1010 (48) | 0 (1130) |

Numbers of individuals at risk and censored events at each time point.

**Figure S1 – Sample formation.**


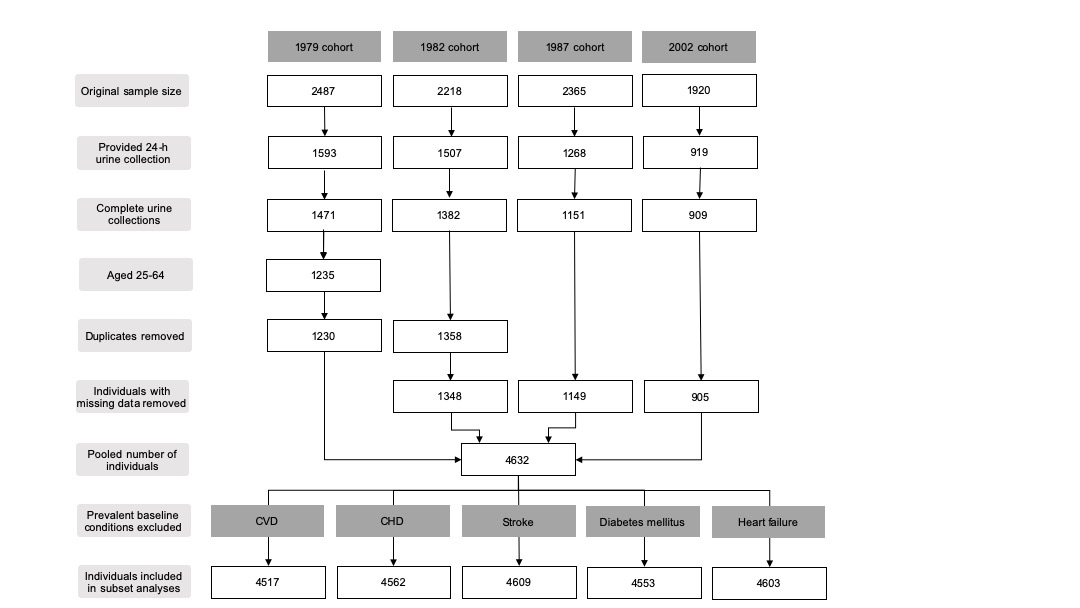


Flow chart depicting the study sample selection.

**Figure S2 – Unadjusted forest plots.**


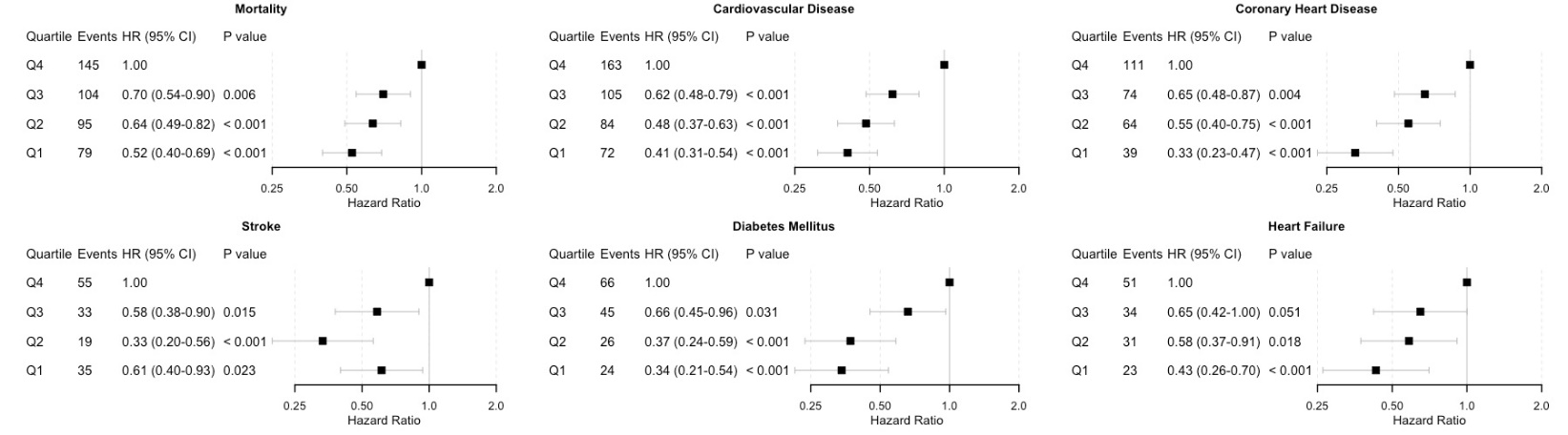


Unadjusted risk of adverse health outcomes by quartiles of 24-h urinary sodium excretion.

**Figure S3 – Unadjusted restricted cubic splines.**


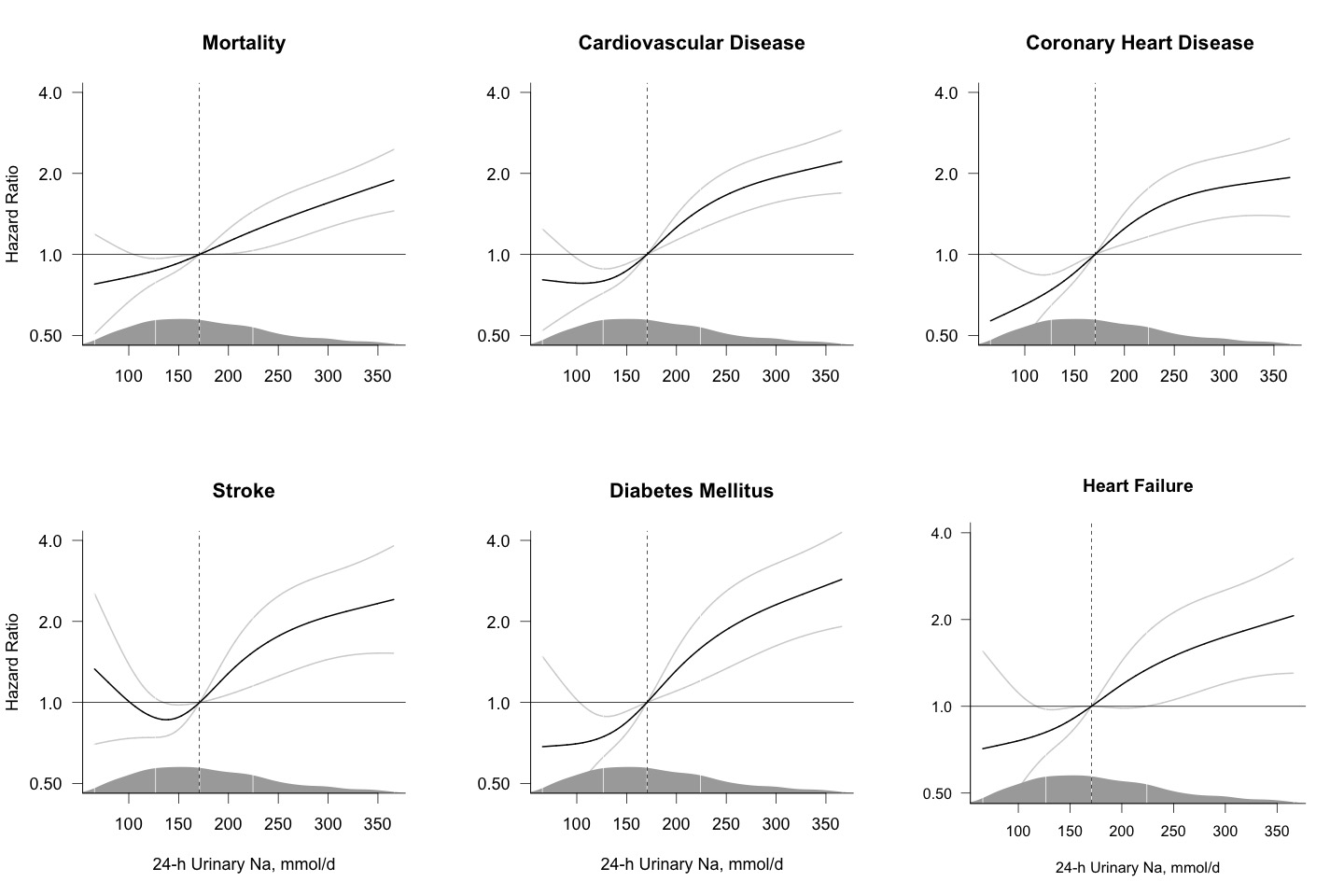


Unadjusted risk of adverse outcomes by 24-hour sodium excretion.

**Figure S4 - Restricted cubic splines with smoking data.**


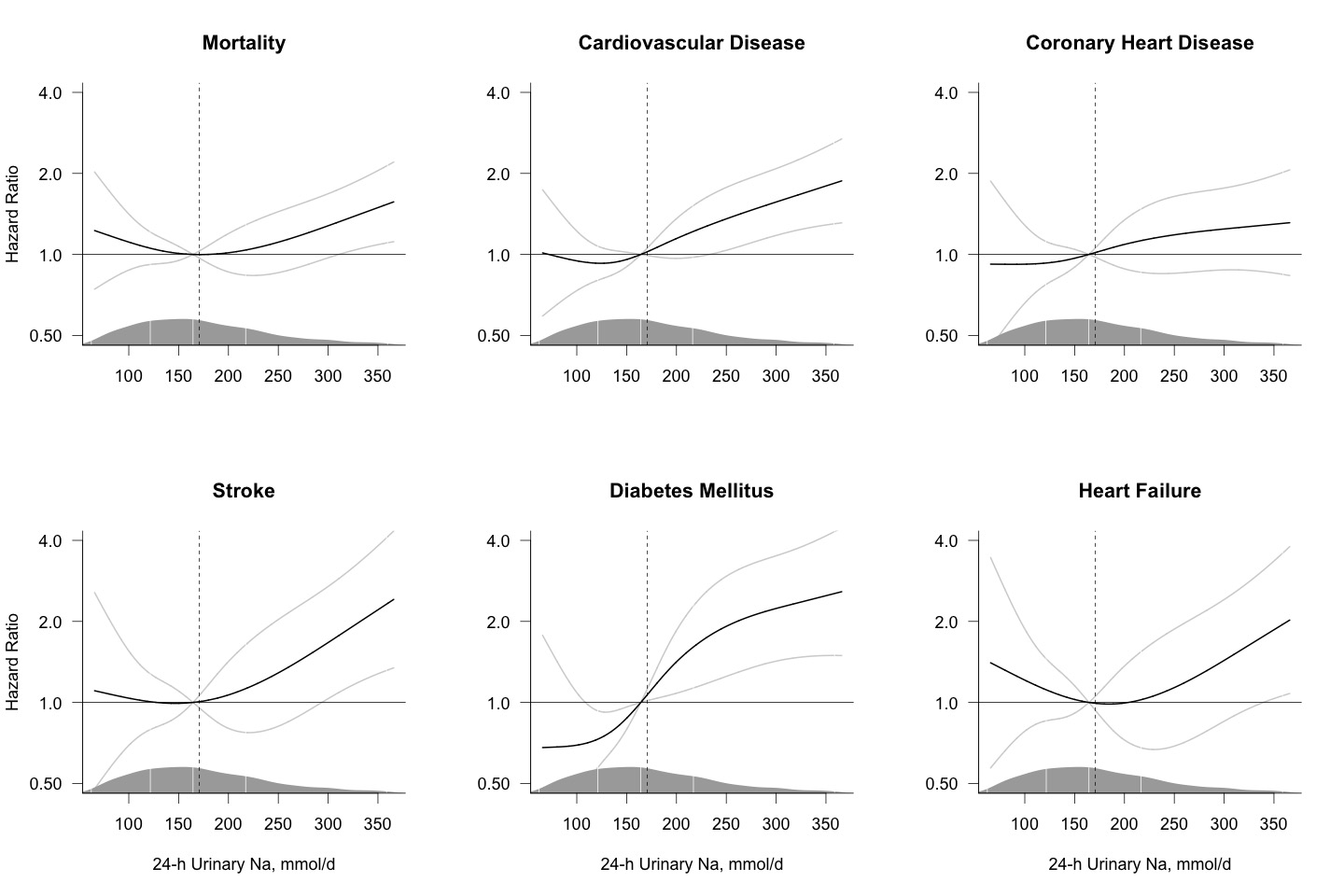


Multivariable-adjusted risk of adverse health outcomes by 24-hour sodium excretion with smoking included as a covariate.

**Figure S5 - Restricted cubic splines with blood pressure baseline data.**


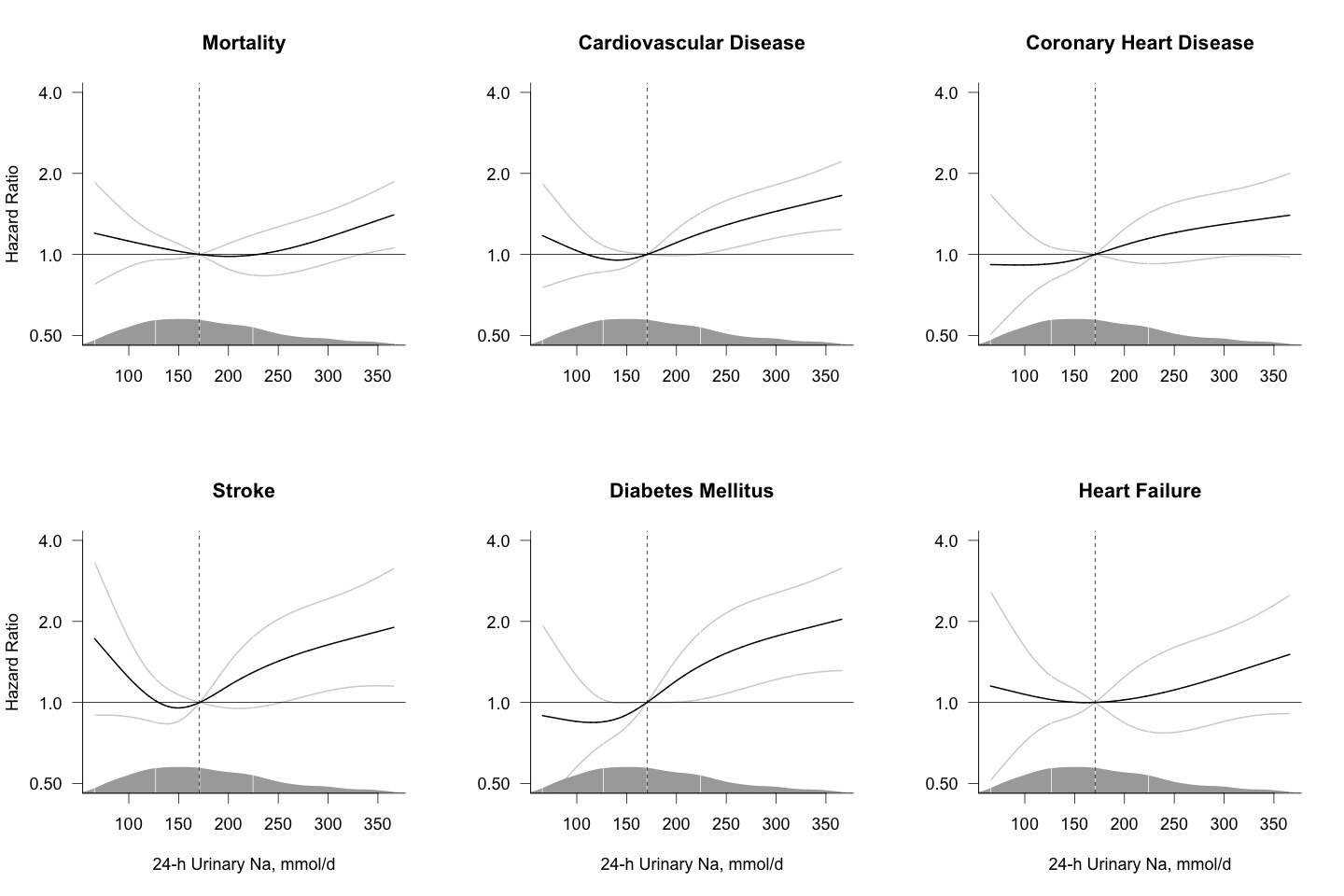


Multivariable-adjusted risk of adverse health outcomes by 24-hour sodium excretion with baseline systolic blood pressure included as a covariate.

**Figure S6 - Subgroup analysis by cohort year for cardiovascular disease with restricted cubic splines.**


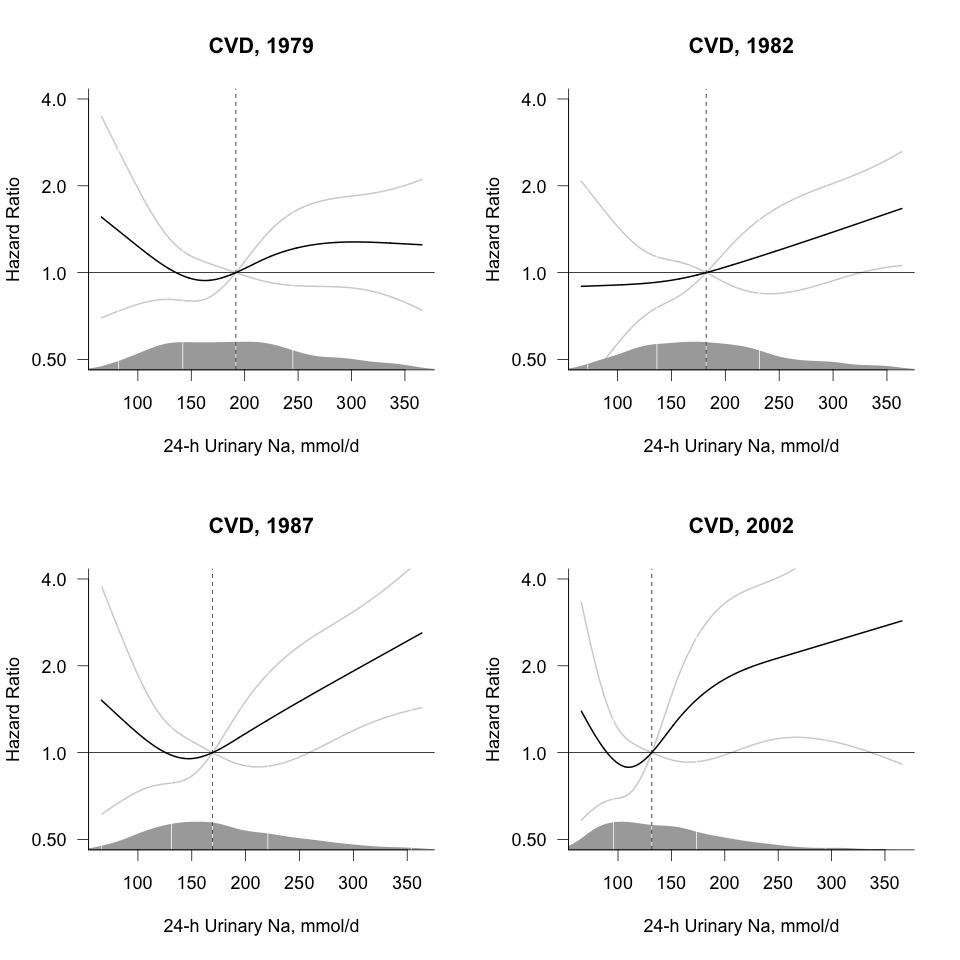


Multivariable-adjusted risk of cardiovascular disease by 24-hour sodium excretion, subgroup analysis by cohort year. Abbreviations: CVD, cardiovascular disease.
